# Elacridar Reverses P-gp-Mediated Drug Resistance in Ovarian Cancer Cells in 2D and 3D Culture Models

**DOI:** 10.3390/ijms262412105

**Published:** 2025-12-16

**Authors:** Piotr Stasiak, Justyna Sopel, Julia Maria Lipowicz, Agnieszka Anna Rawłuszko-Wieczorek, Karolina Sterzyńska, Jan Korbecki, Radosław Januchowski

**Affiliations:** 1 Institute of Biological Sciences, University of Zielona Góra, 65-417 Zielona Góra, Poland; 2 The Doctoral School of Exact and Technical Sciences, University of Zielona Góra, 65-417 Zielona Góra, Poland; 3 Institute of Health Sciences, Collegium Medicum, University of Zielona Góra, 65-417 Zielona Góra, Polandj.korbecki@inz.uz.zgora.pl (J.K.); 4 Department of Histology and Embryology, Doctoral School, Poznan University of Medical Sciences, 61-701 Poznań, Poland; 5 Department of Histology and Embryology, Poznan University of Medical Sciences, 61-701 Poznań, Poland

**Keywords:** ovarian cancer, multidrug resistance, elacridar, P-glycoprotein (P-gp), 3D cell culture model

## Abstract

Multidrug resistance (MDR) remains a major obstacle in the treatment of ovarian cancer. MDR is often mediated by the overexpression of ATP-binding cassette (ABC) transporters, such as P-glycoprotein (P-gp) and Breast Cancer Resistance Protein (BCRP). In this study, we evaluated the ability of elacridar, a dual P-gp and BCRP inhibitor, to overcome MDR in W1, an ovarian cancer cell line sensitive to Paclitaxel (PAC) and its PAC-resistant variants. Cells were cultured under both two-dimensional (2D) and three-dimensional (3D) conditions to account for differences in tumor-like microenvironments. The *MDR1* gene and P-gp protein expression were determined for the analyzed model; P-gp activity was measured by flow-cytometry and fluorescent observation, with and without elacridar. The MTT tests were carried out to evaluate how elacridar, combined with chemotherapeutics, affects cell viability. Our results demonstrate that elacridar effectively inhibited transporter activity and increased cellular sensitivity to PAC and DOX. The inhibitory effect was observed in both 2D and 3D cultures, although the re-sensitization effect in 3D conditions was less pronounced, reflecting the complexity of tumor-specific resistance mechanisms. These findings highlight elacridar as a promising compound for reversing MDR in ovarian cancer and emphasize the importance of 3D models in preclinical drug evaluation. Further studies in advanced in vitro and in vivo models are required to assess the potential of elacridar better.

## 1. Introduction

Ovarian cancer is the eighth most common cancer diagnosed in women. In 2020, it caused 4.7% of cancer deaths. Moreover, it is one of the most common gynecological cancers, and ranks third in terms of mortality, right after cervical and uterine cancer [1]. The reason for the high mortality rate is the late detection of cancer, which is a result of the initial stages being asymptomatic. Most cases are diagnosed in advanced stages, specifically stages III and IV, according to the FIGO classification, which accounts for 85% of diagnosed cases. Another cause of high mortality is low treatment effectiveness caused by primary or acquired resistance to chemotherapy [1,2,3].

Among ovarian cancers, epithelial ovarian cancer (EOC) is responsible for 90% of ovarian cancer cases. This type of cancer is characterized by a high degree of heterogeneity, which contributes to the histological and genetic complexity of this tumor. EOC is divided into two types that differ in tumor progression and genetic mutations. Type I tumors are low-grade tumors arising from endometriosis or borderline tumors, which include endometrioid, clear cell, mucinous, and low-grade serous carcinoma. Type II tumors are high-grade tumors with unidentified precursors, which include high-grade serous carcinoma, carcinosarcoma, and undifferentiated carcinoma [3,4].

The primary treatment for EOC involves cytoreductive surgery combined with chemotherapy [5]. The first line treatment comprises chemotherapy based on platinum drugs and taxane compounds (Paclitaxel—PAC). Depending on the response to platinum compounds, patients are divided into four main groups: platinum sensitive, partially platinum-sensitive, platinum-resistant, or platinum refractory [6]. Unfortunately, as many as 70% of patients experience the development of resistance and disease relapse. Patients with EOC resistant to platinum compounds are treated in the second line of chemotherapy with liposomal doxorubicin (DOX), gemcitabine, or topotecan (TOP) [3,7].

Low response rate to chemotherapy results from the presence of different mechanisms of drug resistance, which can be intrinsic or can develop during chemotherapy. Functionally, the mechanisms of drug resistance can be divided into those specific to cancer tissue and cancer cells specific ones [8].

The cancer-tissue-specific resistance is related to the tumor microenvironment and organization of the tumor tissue: high cell density in the tumor, the presence of blood vessels, the presence of other cells, like Cancer-Associated Fibroblasts (CAF), and non-cellular components (extracellular matrix (ECM), ECM-remodeling enzymes, and growth factors) [9,10]. Increased expression of ECM components is observed in chemotherapy-resistant cancer cells, which suggests an important role of ECM in drug resistance [11,12]. High tumor tissue density and the presence of ECM components may reduce drug availability by limiting drug diffusion or binding [13,14]. Moreover, ECM components can interact with cellular receptors (mainly integrins) and activate pro-survival signaling pathways, which is defined as cell adhesion-dependent drug resistance (CAM-DR). This resistance may also affect the upregulation of ABC drug transporters [15,16].

The active removal of cytotoxic agents from cancer cells is the most important mechanism of resistance at the cellular level. This activity results from the overexpression of ATP-binding cassette (ABC) transport proteins. ABC transporters have low substrate specificity and are thus able to remove different drugs, which are not related in chemical structure, from the cells. This phenomenon is designated as multidrug resistance (MDR) [17]. One of the most important ABC transporters is the MRP2 protein (ABCC2, encoded by the *ABCC2* gene), which is responsible for resistance to several drugs, including cisplatin (CIS). BCRP (ABCG2) is another MDR protein that plays an important role in resistance to various drugs, such as anthracyclines, mitoxantrone, and TOP. The most important ABC transporter is P-glycoprotein (P-gp/ABCB1/MDR1, encoded by the *MDR1* gene), which is responsible for the transport of various compounds [18,19].

P-gp belongs to the eukaryotic ABC superfamily of membrane transporters and is classified as a surface ATPase [20]. It is encoded by the *MDR1* gene, which is located on chromosome 7q2, and consists of 29 exons [21]. The protein consists of 1280 amino acids and has a molecular mass between 140 and 150 kDa. The molecular mass of P-gp increases to 170 kDa after N-glycosylation of amino acids 91, 94 and 99 [22,23]. P-gp, as a transmembrane protein, has 12 transmembrane domains (TMD), two nucleotide-binding domains (NBD) and two ATP-binding domains located in the intracellular part of the protein. This protein does not have a well-defined ligand-binding pocket [24].

In physiological conditions, due to its transport capabilities, P-gp is expressed in epithelial cells, mainly with secretory and excretory functions, e.g., epithelium lining the trachea, mammary glands, prostate, biliary ducts, kidneys, intestine, and brain–blood barrier [25]. This protein plays an important role in liver cells, where it prevents xenobiotics or harmful chemicals from entering the cells [24]. P-gp is also responsible for the secretion of various endogenous and exogenous compounds into the bile [26]. Expression of P-gp protein in the intestine may reduce the bioavailability of some drugs [27].

Unfortunately, P-gp protein is also expressed in cancer cells. In some cancers, the expression can be primary. However, in most cases, expression of P-gp protein increases after chemotherapy [28,29]. High P-gp expression is observed in cancers such as breast, colon, lung, prostate, and ovarian cancer, leukemia, or glioblastoma [28]. Expression of P-gp and other ABC proteins in cancer stem cells (CSCs) also appears to influence the transport of signaling molecules contributing to tumorigenesis [30].

Overexpression of ABC transporters, including P-gp, in cancer cells causes the development of MDR and is associated with poorer patient prognosis [24,25]. The P-gp protein is responsible for removing many chemotherapeutic agents used in cancer treatment from the cells, such as anthracyclines (DOX, daunorubicin, epirubicin), actinomycin D, etoposide, mitomycin C, mitoxantrone, vinblastine, vincristine, taxanes (e.g., PAC), and TOP used in ovarian cancer treatment [24]. Among P-gp substrates used in chemotherapy of ovarian cancer, PAC stabilizes microtubules, preventing cell mitosis, in contrast, DOX intercalates between DNA’s double helix base pairs, resulting in the inhibition of replication and transcription [31,32].

The acquisition of MDR by tumors significantly complicates the treatment and, consequently, patient prognosis. Therefore, attempts are being made to overcome resistance to drugs. Among many different targets, ABC transporters are very promising. Compounds that inhibit ABC transporters or compete with chemotherapeutics for transport through ABC proteins are particularly sought after [20].

The inhibition of ABC protein activity causes increased accumulation of therapeutic agents inside cancer cells, which results in their concentration reaching therapeutic values [20]. One of the P-gp inhibitors is elacridar, which is a third-generation inhibitor of ABC transporters. The third-generation inhibitors are compounds synthesized specifically for the optimal inhibition of a given protein [24]. This inhibitor is a derivative of acridone carboxamide, a tricyclic acridine-based drug used in chemotherapy. It is also known as GG918, GF120918, or N-{4-[2-(1,2,3,4-tetrahydro-6,7-dimethoxy-2-isoquinolinyl)-ethyl]-phenyl}-9,10-dihydro-5-methoxy-9-oxo-4-acridine carboxamide. The molar mass of this compound is 563.64 g/mol [33]. Elacridar binding to P-gp most likely occurs in the transmembrane domains and competes for the binding site of [3H]azidopine [28]. This inhibitor is not specific to P-gp. It can also inhibit the activity of BCRP, another ABC transporter [34,35].

The effect of elacridar on drug sensitivity has been tested in many cancer cell lines and resulted in sensitization of cancer cells to drug treatment [36]. Drug-resistant prostate cancer cells with concomitant positive P-gp expression have been shown to regain sensitivity to docetaxel (DTX) when treated with elacridar [37]. Zhou et al. showed that elacridar sensitized highly resistant leukemia cells to daunorubicin and mitoxantrone [38]. Also, hepatoblastoma cells with increased P-gp expression increased their sensitivity to DOX after treatment with this inhibitor [39]. Elacridar was used to treat PAC-resistant human gastric cancer cells, resulting in sensitization of these cell lines to PAC [40]. This inhibitor also enhanced the efficacy of TOP in an ovarian cancer cell line and in cultured canine kidney cells overexpressing BCRP [35,41].

In preclinical studies, elacridar was studied in mice, rats, dogs, and monkeys [42,43]. The aim of these studies was to evaluate pharmacokinetic parameters, which showed good absorption and no effect on P450 enzymes [43]. Additionally, oral administration of taxanes with elacridar increased the concentrations of PAC and DTX in mouse plasma [43]. Jonker et al. showed that the bioavailability of TOP was increased by co-administration of elacridar in mice [44].

The use of elacridar in DOX co-treatment resulted in the reversal of resistance to DOX in P388/DOX tumors in a mouse xenograft model [45]. Moreover, administration of elacridar with levatinib to mice with hepatocellular carcinoma resulted in a better treatment response than administration of levatinib alone [46]. Omori et al. showed that combination therapy with elacridar and a topoisomerase inhibitor, Etoposide inhibits the growth of small cell lung cancer tumors in vivo [47]. Co-administration of elacridar and sunitinib inhibited tumor growth in sunitinib-resistant metastatic clear cell renal cancer in chicken chorioallantoic membrane and mouse models [48].

In clinical trials, the oncology patients with solid tumors were treated with a combination of DOX and elacridar. Increased exposure to doxorubicinol (a metabolite of DOX) was observed in patients treated with co-therapy compared to patients treated with DOX alone [49]. In other clinical studies, elacridar has been shown to significantly increase the systemic exposure to oral PAC in oncology patients [27]. Elacridar has also been shown to increase the bioavailability of TOP in cancer patients [44].

In the presented studies, PAC-sensitive and -resistant ovarian cancer cell lines were used. Previously, PAC-resistant cell lines W1PR1, W1PR1-C7, and W1PR2 were derived from the drug-sensitive W1 cell line. All PAC-resistant lines used in this study are characterized by high levels of P-gp expression [50,51] and are capable of growing in 2D and 3D cell culture conditions. The 2D model is a common model used in research. However, it does not reflect the conditions prevailing in the tumor, such as tumor structure, tumor cell packing, or overexpression of ECM molecules, which affect the treatment effect by lower drug diffusion. The 3D model allows for a better, although still not perfect, representation of the conditions inside the tumor [49]. Previous studies show that in a 3D model, cells exhibit greater resistance to chemotherapeutic drugs than those cultured in a monolayer [52,53,54].

The aim of the following studies was to investigate whether inhibition of P-gp activity by elacridar affects the resistance of cancer cells to cytotoxic drugs. To better understand the elacridar effect on drug resistance, we elucidated in 2D cultures a 3D model of cancer cell growth. This work provides a comprehensive analysis of P-gp activity, expression, and inhibition in PAC-sensitive and-resistant ovarian cancer cells.

## 2. Results

### 2.1. Characterization of MDR1 Gene and P-gp Protein Expression in W1 Cell Line and PAC-Resistant Cell Lines

**Figure 1:**
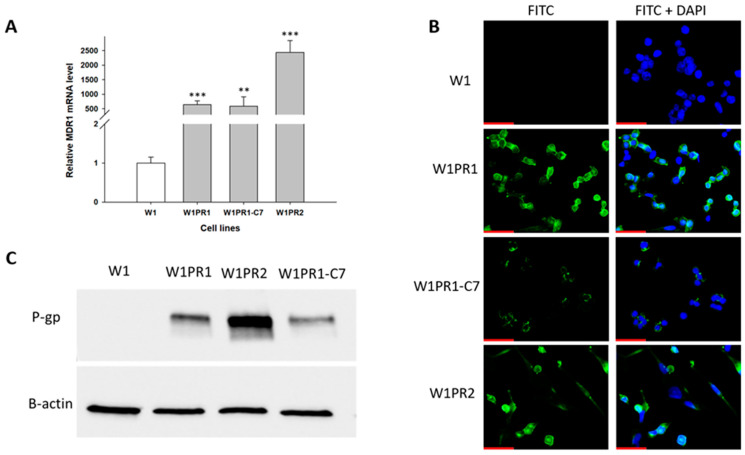
(**A**) Quantitative PCR analysis of *MDR1* mRNA levels in W1 cells and PAC-resistant sublines. The figure shows relative transcript levels in resistant variants (gray bars) compared with the paternal sensitive line (white bar), which was set to a value of 1. Statistical significance indicated as ** *p* < 0.01 and *** *p* < 0.001. (**B**) Immunofluorescent detection of P-gp in W1 and PAC-resistant sublines. P-gp was visualized using an anti-P-gp primary antibody followed by an Alexa Fluor^®^488-conjugated secondary antibody (green). Nuclei were counterstained with DAPI (blue). Images were captured using a 40× objective. Scale bar = 50 μm (red). (**C**) Analysis of P-gp protein levels in monolayer cultures of W1 and PAC-resistant cells. Protein extracts were resolved on 7% PAGE gels, transferred to nitrocellulose membranes, and probed with either primary Ab or HRP-conjugated secondary Ab. A primary anti-β Ab was used as a loading control for the cell lysates.

To study the potential of elacridar to reverse chemoresistance, we selected a model of the W1 ovarian cancer cell line and its PAC-resistant derivative. As the MDR1/P-gp overexpression plays a main role in PAC-resistance, we compared the expression of the *MDR1* gene and P-gp protein in our model. *MDR1* gene expression was determined using Q-PCR analysis, which showed a statistically significant increase in expression of this gene in PAC-resistant cell lines compared to sensitive cell lines (Figure 1A). We observed over 500-fold higher expression of the *MDR1* gene in the W1PR1 cell line (*p* < 0.001) and WIPR1-C7 cell line (*p* < 0.01) and about 2000-fold higher expression in the W1PR2 cell line (*p* < 0.001) than in sensitive cells.

The expression of P-gp protein in all cell lines was checked using the Western blot method (Figure 1B). We observed bands corresponding to P-gp protein in PAC-resistant cell lines, but they were not present in the W1 drug-sensitive cell line. The highest amount of P-gp protein was detected in the W1PR2 cell line.

Immunofluorescence staining was performed to confirm the presence of P-gp protein (Figure 1C). This assay confirmed the presence of P-gp protein in resistant cell lines with a clear localization of P-gp protein in the cell membrane. However, P-gp protein was not detected in the sensitive W1 cell line.

### 2.2. Analysis of P-gp Activity

**Figure 2:**
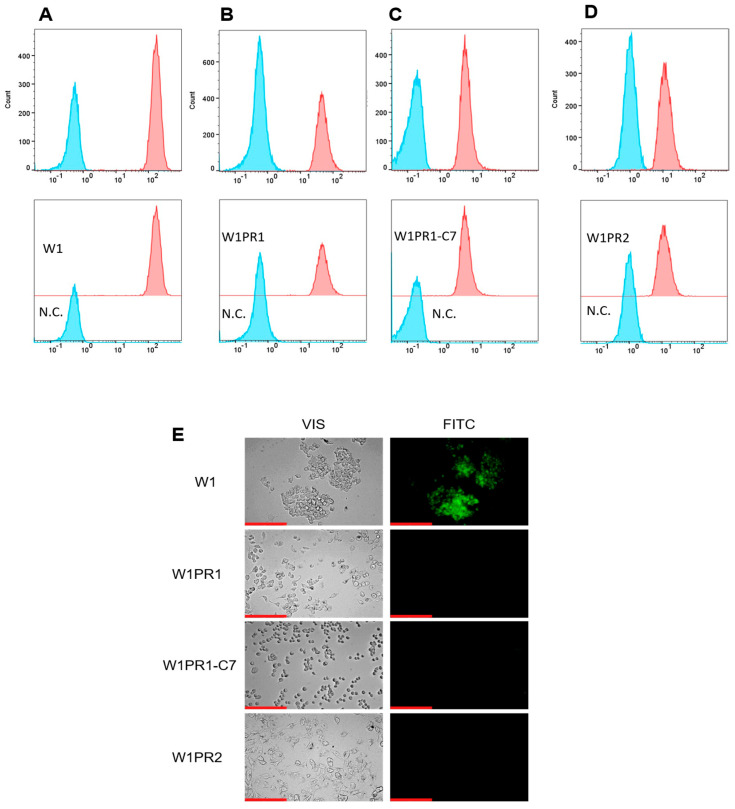
Flow cytometry analysis of intracellular accumulation of Rhodamine 123 (Rho1) accumulation (red) in the drug-sensitive W1 cell line (**A**) and PAC-resistant variants: W1PR1 (**B**), W1PR1-C7 (**C**), and W1PR2 (**D**), compared with their respective negative controls (N.C.) lacking Rh123 (blue). Inverted fluorescence microscopy imaging showing Rho123 retention (green) in the paternal W1 cells and the corresponding resistant sublines W1PR1, W1PR1-C7, and W1PR2 (**E**). Images were captured using a 40× objective. Scale bar = 200 µm (red).

P-gp protein transport activity in W1 and PAC-resistant cell lines was examined by determining the accumulation of Rhodamine 123 (Rho123) using flow cytometry. The fluorescence intensity of accumulated Rho123 in PAC-resistant cell lines was lower than that in the sensitive cell line (Figure 2A–D). The lowest accumulation was observed in the W1PR2 and W1PR1-C7 lines, which is due to greater transport of Rho123 out of the cells. A slightly higher accumulation of Rho123 was observed in the W1PR1 cell line, but it is lower than in the PAC-sensitive line.

The accumulation of Rh123 in cells was also investigated using an inverted fluorescence microscope (Figure 2E). The intravital imaging of PAC-resistant cell lines did not show Rho123 accumulation. In contrast, in the W1 cell line, we observed a clear Rho123 fluorescence.

### 2.3. Characterization of Drug Resistance in the W1 Cell Line and PAC-Resistant Cell Lines

As we determined increased expression and activity of P-gp drug transporter in PAC-resistant cell lines, we were interested in whether P-gp expression/activity is reflected by the level of drug resistance. Thus, we used drugs that are the substrates of P-gp PAC and DOX, as well as CIS, a drug that is not a P-gp substrate. For all the cell lines, we determined the drug concentration that caused a 50% reduction in cell viability (IC_50_). All PAC-resistant cell lines showed a significant increase in PAC resistance. In the W1PR1 cell line, we observed a 616-fold increase in IC_50_ value, compared to the W1 cell line (IC_50_ = 1960 ng/mL vs. IC_50_ = 3.18 ng/mL). Lower resistance was demonstrated in the W1PR1-C7 line, which showed a 377-fold increase in resistance (IC_50_ = 1199 ng/mL vs. IC_50_ = 3.18 ng/mL), compared to the sensitive cell line. The highest PAC-resistance was observed in the W1PR2 cell line, with a 1064-fold increase in resistance in comparison to the W1 cell line (IC_50_ = 3384 ng/mL vs. IC_50_ = 3.18 ng/mL) (Table 1).

DOX was the next cytotoxic drug that we analyzed. The IC_50_ value for the sensitive cell line was 32.07 ng/mL. In all resistant cell lines, we observed an increased resistance to DOX compared to the W1 cell line. DOX resistance increased 215-fold for the W1PR1 line (IC_50_ = 6904 ng/mL) and 86-fold for the W1PR1-C7 cell line (IC_50_ = 2769 ng/mL). The highest IC_50_ was determined in the W1PR2 cell line, which was 7836 ng/mL, which resulted in 244-fold higher resistance compared to the sensitive cell line (Table 1).

The last cytotoxic agent tested was CIS, the drug used in the first line of ovarian cancer chemotherapy. We did not observe significant differences in resistance between the investigated cell lines. For the W1 cell line, the IC_50_ was determined to be 19,741 ng/mL. PAC-resistant lines showed IC_50_ of 21,566 ng/mL (W1PR1), 14,118 ng/mL (W1PR1-C7), and 16,779 ng/mL (W1PR2) (Table 1).

### 2.4. MTT Analyses of Elacridar Effect on Resistance to Cytotoxic Drugs

**Figure 3:**
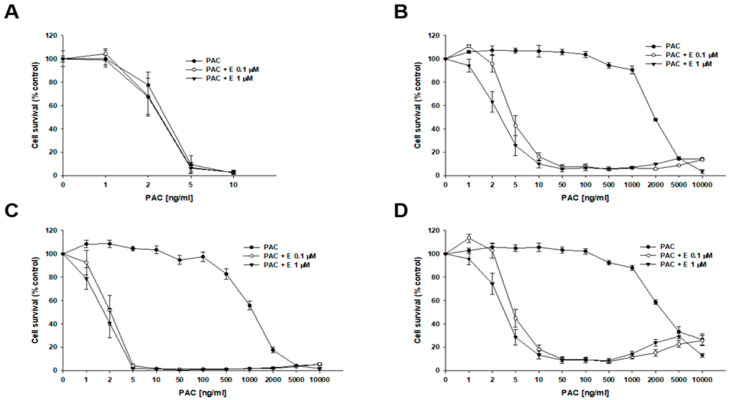
Elacridar (E) enhances the sensitivity of PAC-resistant cell lines to PAC in vitro. The PAC-sensitive W1 line (**A**) and the PAC-resistant variants W1PR1 (**B**), W1PR1-C7 (**C**), and W1PR2 (**D**) were seeded into 96-well plates and exposed for 72 h to. PAC alone or to PAC in combination with E at final concentrations of 0.1 or 1 µM. Following the 72 h treatment period, cell viability was assessed using the MTT assay. Results are presented as the percentage of untreated controls (mean ± SEM).

To determine whether inhibiting P-gp activity would increase the sensitivity of cells to cytotoxic drugs, we used a selective P-gp inhibitor, elacridar, at concentrations of 0.1 µM and 1 µM. The first drug analyzed was PAC (Figure 3, Table 2). In the W1 drug-sensitive cell line, we did not observe any effect of elacridar on PAC-resistance (Figure 3A): IC_50_ = 3.18 ng/mL without elacridar vs. IC_50_ = 2.7 ng/mL with 0.1 µM elacridar and IC_50_ = 2.65 ng/mL with 1 µM elacridar. In all PAC-resistant cell lines, we observed a strong effect of elacridar treatment on PAC resistance. We observed the effect of elacridar in both concentrations used on PAC resistance, starting from 2 ng/mL in the W1PR1 cell line (Figure 3B) and from 1 ng/mL in the W1PR1-C7 cell line, respectively (Figure 3C). This resulted in a decrease in IC_50_ value. In the W1PR1 line, we observed a 404-fold decrease in IC_50_ value in the presence of 0.1 μM elacridar (IC_50_ = 4.85 ng/mL vs. IC_50_ = 1960 ng/mL) and a 607-fold decrease in IC_50_ value in the presence of 1 µM elacridar (IC_50_ = 3.23 ng/mL vs. IC_50_ = 1960 ng/mL). In the W1PR1-C7 cell line, we observed a 538-fold decrease in IC_50_ value (IC_50_ = 2.29 ng/mL vs. IC_50_ = 1199 ng/mL in the presence of 0.1 µM elacridar) and a 620-fold decrease in IC_50_ value in the presence of 1 µM elacridar (IC_50_ = 1.93 ng/mL vs. IC_50_ = 1199 ng/mL). In the W1PR2 cell line, a clear effect of elacridar in the concentration of 0.1 µM was observed in PAC concentration of 5 ng/mL and higher, and 1 µM of elacridar in the PAC concentration of 2 ng/mL and higher (Figure 3D). This was reflected by the 650-fold decrease in IC_50_ value in elacridar concentration of 0.1 µM (IC_50_ = 5.20 ng/mL vs. IC_50_ = 3384 ng/mL) and a 945-fold decrease in IC_50_ value for elacridar in concentration of 1 µM (3.58 ng/mL vs. IC_50_ = 3384 ng/mL).

**Figure 4:**
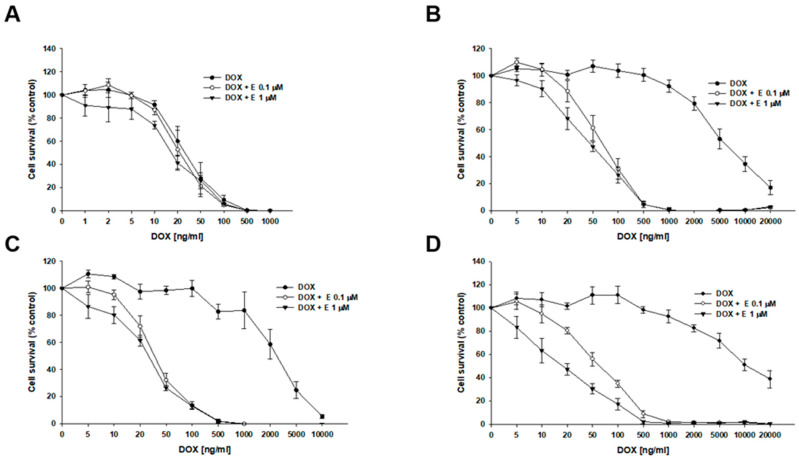
Elacridar (E) increases DOX sensitivity in PAC-resistant cell lines in vitro. The PAC-sensitive W1 line (**A**) and PAC-resistant sublines W1PR1 (**B**), W1PR1-C7 (**C**), and W1PR2 (**D**) were seeded into 96-well plates and exposed for 72 h to DOX alone or DOX combined with E at final concentrations of 0.1 or 1 μM. After treatment, cell viability was measured using the MTT assay and expressed as a percentage of untreated controls (mean ± SEM).

Next, we investigated the effect of elacridar on resistance to another P-gp substrate—DOX (Figure 4, Table 3). In the W1 cell line, we observed a slight, though not statistically significant, decrease in IC_50_ value after elacridar treatment (IC_50_ = 32.07 ng/mL vs. IC_50_ = 25.0 ng/mL (0.1 µM E) vs. IC_50_ = 21.6 ng/mL (1 µM E). For PAC-resistant cell lines, the elacridar treatment resulted in a reduction in resistance to DOX, and the effect was clearly dependent on the concentration used. Namely, in the W1PR1 cell line, elacridar in concentrations of 0.1 µM or 1 µM resulted in 82-fold (IC_50_ = 6904 ng/mL vs. IC_50_ = 84.6 ng/mL) or 152-fold (IC_50_ = 6904 ng/mL vs. IC_50_ = 45.5 ng/mL) decrease in IC_50_ value, respectively, compared to DOX alone. The W1PR1-C7 line showed a 77-fold and 98-fold decrease in IC_50_ value in the presence of 0.1 µM or 1 µM elacridar, respectively (IC_50_ = 2769 ng/mL vs. IC_50_ = 36.1 ng/mL vs. IC_50_ = 28.4 ng/mL). The most significant effect of elacridar on DOX resistance was observed in the W1PR2 cell line, where we observed 123-fold and 436-fold reduction in IC_50_ value in comparison to cells treated with DOX only (IC_50_ = 7836 ng/mL vs. IC_50_ = 63.8 ng/mL vs. IC_50_ = 18.0 ng/mL).

**Figure 5:**
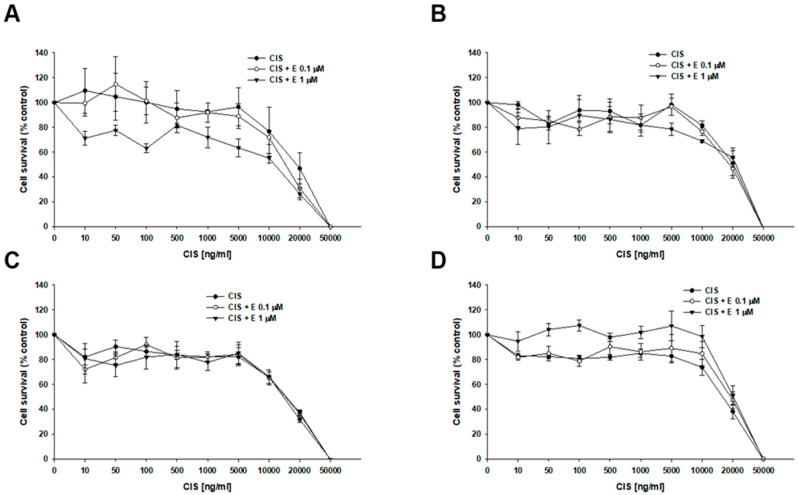
Response of PAC-sensitive and PAC-resistant cell lines to CIS in the presence and absence of elacridar (E) in vitro. The PAC-sensitive W1 line (**A**) and the PAC-resistant sublines W1PR1 (**B**), W1PR1-C7 (**C**), and W1PR2 (**D**) were seeded into 96-well plates and exposed for 72 h to CIS alone or CIS combined with E at final concentrations of 0.1 or 1 μM. After treatment, cell viability was assessed using the MTT assay and expressed as a percentage of the untreated controls (mean ± SEM).

The effect of elacridar and CIS co-treatment was also analyzed (Figure 5). In both the sensitive and PAC-resistant cell lines, no statistically significant changes in cell viability were observed during co-treatment. For the W1 cell line, the IC_50_ was determined to be 19,741 ng/mL (CIS alone), 16,355 ng/mL (CIS + 0.1 µM E), and 12,752 ng/mL (CIS + 1 µM E). The IC_50_ determined for resistant cell lines was: W1PR1—IC_50_ = 21,566 ng/mL vs. IC_50_ = 20,099 ng/mL vs. IC_50_ = 22,470 ng/mL; W1PR1-C7—IC_50_ = 1418 ng/mL vs. IC_50_ = 14,906 ng/mL vs. IC_50_ = 14,998 ng/mL; W1PR2—IC_50_ = 16,779 ng/mL vs. IC_50_ = 20,541 ng/mL vs. IC_50_ = 21,291 ng/mL (Table 4).

### 2.5. Analysis of P-gp Expression in Elacridar Treated Cell Lines

**Figure 6:**
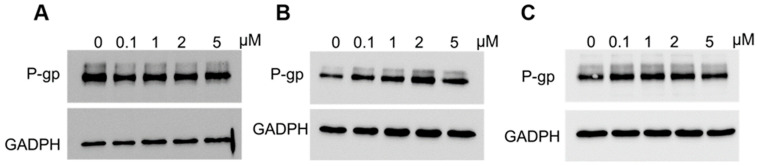
Analysis of P-gp protein levels in the W1PR1 (**A**), W1PR1-C7 (**B**), and W1PR2 (**C**) cell lines cultured as monolayers following 72 h of elacridar treatment at 0.1, 1, 2, or 5 µM. Cellular proteins were resolved on 7% PAGE gels, transferred onto nitrocellulose membranes, and probed with the appropriate primary Ab or HRP-conjugated secondary Ab. A primary anti-GADPH Ab was used as a loading control.

Next, we performed an analysis of P-gp protein expression in PAC-resistant cells lines following elacridar treatment (Figure 6). Cancer cells were treated with elacridar at different concentrations (0, 1, 2 or 5 µM) for 72 h. After this time, P-gp protein levels were analyzed by Western blot. The level of the tested protein did not change in any PAC-resistant line after elacridar treatment.

### 2.6. Analysis of P-gp Activity in Cell Lines Treated with Elacridar

**Figure 7:**
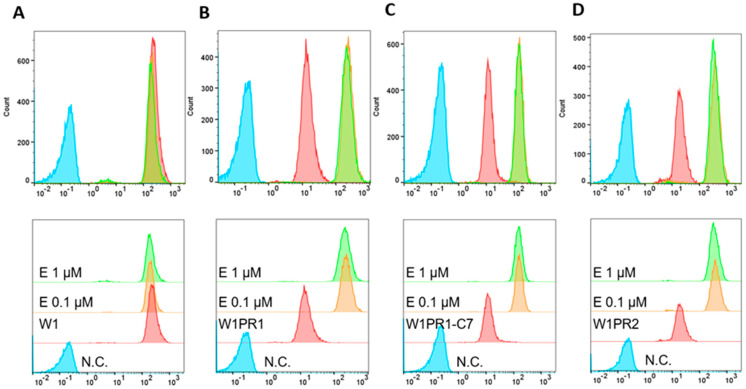
Flow cytometry analysis of Rhodamine123 (Rho123) accumulation in the PAC-sensitive W1 line (**A**) and PAC-resistant sublines W1PR1 (**B**), W1PR1-C7 (**C**), and W1PR2 (**D**). Fluorescence profiles are shown for untreated cells (red) and for cells exposed to elacridar (E) at 0.1 µM (orange) or 1 µM (green). Negative controls lacking Rho123 (N.C) are marked in blue.

To check the activity of P-gp protein after treatment of cancer cells with elacridar, Rho123 fluorescence analysis using flow cytometry was performed. Rho123 fluorescence intensity assay in cells showed that elacridar does not affect the accumulation of the dye in PAC-sensitive cells (Figure 7A). In PAC-resistant cell lines, treatment with elacridar in concentration of 0.1 µM and 1 µM resulted in increased accumulation of the Rho123 dye with very similar effect in both concentration used (Figure 7B–D).

**Figure 8:**
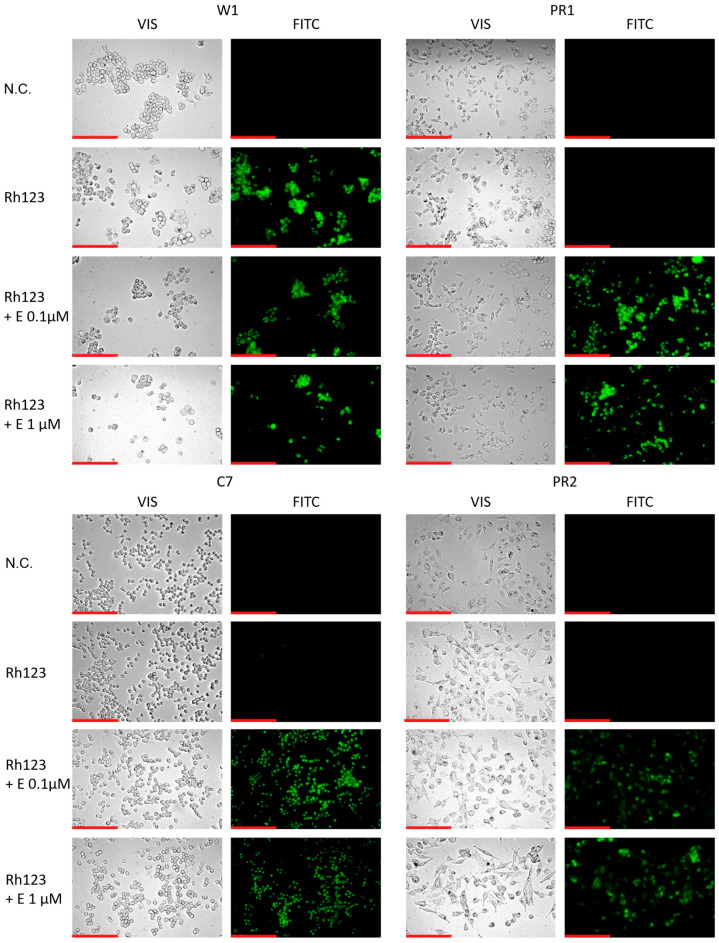
Fluorescence microscopy visualization of Rh123 retention (green) in PAC-sensitive W1 line and in PAC-resistant sublines: W1PR1, W1PR1-C7, and W1PR2, with and without elacridar (E) at 0.1 µM and 1 µM. Images were acquired using a 40× objective. Scale bar = 200 µm (red).

To further confirm the effect of elacridar on P-gp activity we analyzed the Rho123 accumulation in live cells using inverted fluorescence microscope. In W1 drug sensitive cell line we observed a similar Rho123 fluorescence in the presence and absence of elacridar in both concentration used. In contrast in all PAC-resistant cell lines Rho123 fluorescence was observed only after elacridar treatment and was independent of elacridar concentration used (Figure 8).

### 2.7. Expression and Activity of P-gp in Sensitive and PAC-Resistant Cancer Cell Lines in 3D Model

**Figure 9:**
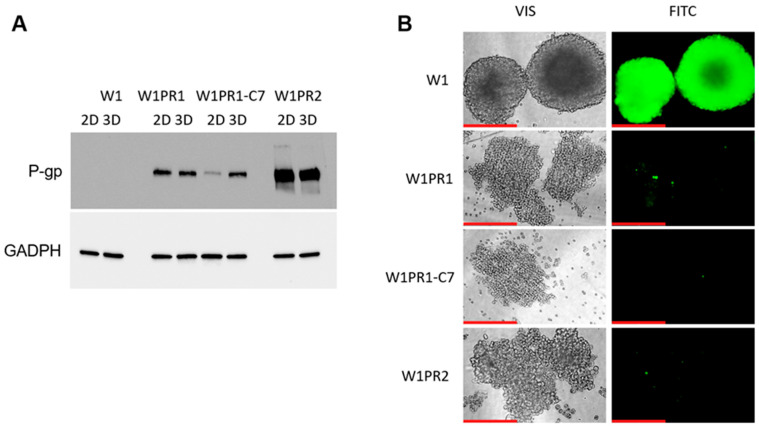
(**A**) P-gp protein levels in W1 and PAC-resistant cell lines cultured either as monolayers (2D) or spheroids (3D). Protein extracts were separated on 7% PAGE gels, transferred to nitrocellulose membranes, and probed with either primary Ab or HRP-conjugated secondary Ab. A primary anti-GADPH Ab was used as a loading control. (**B**) Fluorescence microscopy images showing Calcein-AM accumulation (green) in 3D spheroids generated from the PAC-sensitive W1 cell line and the PAC-resistant sublines W1PR1, W1PR1-C7, and W1PR2. Images were taken using 20× objective. Scale bar = 200 μm (red).

To further investigate the effect of elacridar on drug resistance, a 3D cell culture model was used. It can be observed that the W1 cell line forms compact spheroids, while the PAC-resistant cell lines form a less compact spheroid structure (Figure 9B). First, the expression and activity of P-gp protein were checked in the studied cell lines. P-gp protein levels were compared in 2D and 3D cell cultures (Figure 9A). The sensitive cell line did not express the tested protein under any conditions. All PAC-resistant cell lines (W1PR1, W1PR1-C7, W1PR2) showed P-gp expression in both culture models used. The W1PR1-C7 cell line showed higher P-gp expression for 3D culture. However, the remaining PAC-resistant cell lines did not show any difference in the level of the tested protein depending on the cell culture model used.

The ability of cancer cells to accumulate Calcein-AM in a 3D model was also tested, which translates into the activity of P-gp proteins (Figure 9B). The dye was accumulated only in the PAC-sensitive cell line sphere. In contrast, in spheroids formed from PAC-resistant cell lines we did not observe CA accumulation.

### 2.8. Analysis of P-gp Activity in Elacridar-Treated Spheroids

**Figure 10:**
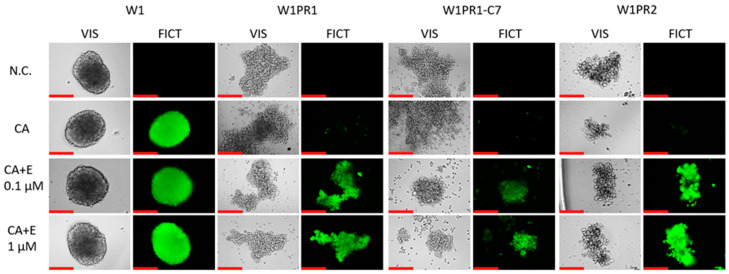
Fluorescence microscopy assessment of Calcein-AM retention (green) in spheroids derived from the PAC-sensitive W1 line and the PAC-resistant W1PR1, W1PR1-C7, and W1PR2 sublines, treater either without or with elacridar (E) at 0.1 µM and 1 µM. Images were obtained using 20× objective. Scale bar = 200 μm (red).

The ability of 3D spheroids to accumulate Calcein-AM after elacridar treatment was tested (Figure 10). In the PAC-sensitive cell line, we did not observe a change in the accumulation of this compound. At both elacridar concentrations, there is no visible change in Calcein-AM accumulation in the spheres formed from the W1 line in comparison to the control. In spheroids generated from PAC-resistant cell lines, no dye accumulation was observed in the control. In the presence of 0.1 µM elacridar, we observed Calcein-AM accumulations in spheroids from all three Pac-resistant cell lines. Increasing the inhibitor concentration to 1 µM did not significantly increase Calcein-AM accumulation.

### 2.9. Analysis of the Effect of Elacridar on Response to Cytotoxic Drug Treatment in a 3D Model

**Figure 11:**
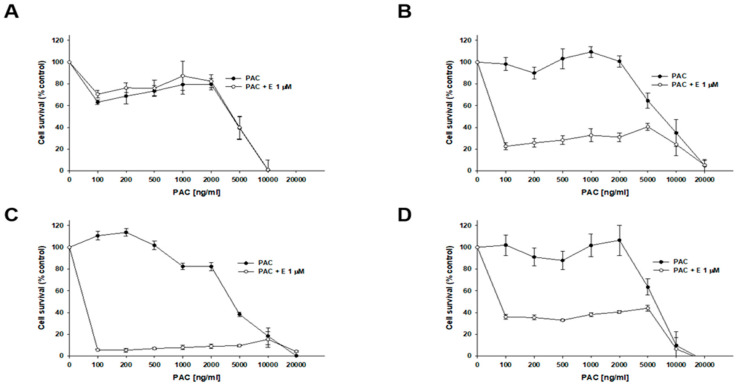
Effect of elacridar (E) on PAC sensitivity in PAC-sensitive and PAC-resistant cell lines under 3D culture conditions. Spheroids were formed from 15,000 (15k) of cells for each line: W1 (**A**), W1PR1 (**B**), W1PR1-C7 (**C**), and W1PR2 (**D**). Spheroids were exposed to PAC or PAC + E at 1 µM. Cell viability was evaluated using the MTT assay and expressed as a percentage of untreated controls (mean ± SEM).

The effect of elacridar on the response of spheroids to PAC treatment was assessed. The cytotoxicity of PAC was checked using the MTT method and determining the IC_50_ for given conditions. Spheroids obtained from 1.5 × 10^4^ cells were treated with 1 µM elacridar and increasing concentrations of PAC for 72 h. In the W1 cell line, no significant changes in IC_50_ values were observed between cells treated with PAC alone (IC_50_ = 4539 ng/mL) and cotreatment with elacridar (IC_50_ = 4597 ng/mL) (Figure 11A, Table 5). However, for PAC-resistant cell lines, the presence of elacridar resulted in a reduction in the IC_50_ value for PAC (Figure 11B,C, Table 5). Namely, for the W1PR1 cell line, the IC_50_ value reduction was the greatest: 129-fold (decrease from IC_50_ = 8405 ng/mL to IC_50_ = 65.3 ng/mL). For the W1PR1-C7 cell line, a decrease in IC_50_ was noted (from IC_50_ = 4187 ng/mL to IC_50_ = 53 ng/mL, which is a 79-fold reduction in value). The IC_50_ value for the co-treated W1PR2 cell line decreased 73.5-fold (decrease from IC_50_ = 5894 ng/mL to IC_50_ = 78.3 ng/mL) (Figure 11D).

## 3. Discussion

Despite the enormous progress of cancer research that has occurred in over past 20 years, EOC statistics and prognoses for patients remain poor [55]. The nonspecific symptoms, late diagnosis, and eventual development of drug resistance continue to be obstacles to successful treatment [56]. As overexpression of ABC transporters plays a significant role in drug resistance, the use of ABC transporter inhibitors to tackle the issue of drug resistance is one of the approaches to improve effective treatment [57].

In our work, we have tested whether elacridar, a third-generation ABC transporter inhibitor, could be considered of use to combat PAC and DOX resistance. To study the impact of elacridar, we used a model of MDR development composed of the drug-sensitive W1 ovarian cancer cell line and its sublines—W1PR1, W1PR1-C7, and W1PR2—developed by the treatment with PAC [51,52].

As PAC and DOX resistance is most often associated with P-gp overexpression [17], we started by analyzing the expression of *MDR1* transcript and P-gp protein expression in our model. We concluded that the expression of *MDR1*/P-gp in PAC-resistant cell lines is much higher compared to the drug-sensitive cell line, and P-gp in drug-resistant cell lines is mainly localized in the plasma membrane, which is consistent with our previous studies [51,58].

To further investigate whether P-gp expression corresponds to increased activity in the researched cells, we have checked the cellular ability to retain the common fluorescent substrate of this protein, Rho123. This compound is a popular fluorone dye used to examine MDR1-related membrane transport in vitro [59]. Both the tests conducted on the Flow Cytometer and under the microscope concluded that Rho123 is accumulated only in the sensitive cell line, whereas it is actively removed by P-gp in all of the PAC-resistant cell lines. This is consistent with our previous works concerning different PAC-resistant ovarian cancer cell lines [60], as well as with the works of other authors [61].

As P-gp transports many cytotoxic drugs, our next step was to assess the resistance of the investigated cell lines to PAC and DOX, a commonly used P-gp substrate and a cytotoxic drug that is not a substrate of this protein—CIS [18]. We noted a significant increase in PAC and DOX resistance among all of the PAC-resistant cell lines when compared to the sensitive W1 cell line, with the lower resistance in the W1PR1-C7 cell line and the higher resistance in the W1PR2 cell line. The level of resistance seems to correlate with P-gp protein expression. A similar correlation was observed by us previously [60]. On the other hand, no significant change in CIS resistance was observed in the tested cell lines. This further confirms that drug resistance in this model is facilitated by P-gp. For further study, we used this model to investigate the effect of elacridar on P-gp activity and drug resistance.

Thus, using the MTT assay, we tested how elacridar in concentrations of 0.1 μM and 1 μM affects the PAC, DOX, and CIS resistance of the investigated cell lines. In case of PAC and DOX, we observed that the addition of elacridar in both concentrations caused a powerful decrease in PAC IC_50_ value to a level similar to those in W1 drug-sensitive cell line, but it did not have a significant effect on PAC or DOX resistance in the W1 cell line. The effect of elacridar was concentration-dependent and increased the higher concentration was used. Similar results were observed by our team previously, in PAC-resistant sublines of A2780 cell culture [60]. In case of CIS resistance, elacridar had no relevant effect, regardless of the concentration used, and this was also observed by our team previously [60]. This was to be expected, as CIS is not a P-gp substrate [55].

In studies on chronic myeloid leukemia (CML) cell lines K562 and LAMA-84, as well as imatinib-resistant cultures, elacridar at a concentration of 0.25 μM effectively inhibited ABC transporter activity and led to a 5- to 10-fold increase in sensitivity to imatinib, as confirmed by flow cytometry [62]. Similarly, in the daunorubicin-resistant human promyelocytic leukemia cell line HL60/DNR, treatment with 0.1 μM elacridar restored drug sensitivity, resulting in a 40-fold reduction in IC_50_ value for daunorubicin and a 57-fold decrease in IC_50_ value for mitoxantrone [38]. In DTX-resistant human prostate cancer cell lines DU-145R and 22RV1, which show positive P-gp expression, the addition of 0.25 μM elacridar reversed resistance to DTX [37]. In non-small cell lung cancer (NSCLC) cell lines resistant to DTX, the combination of 0.25 μM elacridar with DTX significantly enhanced drug sensitivity, with IC_50_ reductions ranging from 29-fold to over 3000-fold, depending on the cell line [63]. For the CIS-resistant ovarian cancer IGROVCDDP cell line characterized by P-gp overexpression, treatment with 0.25 μM elacridar increased sensitivity to multiple P-gp substrates—including DTX, PAC, epirubicin, and vinblastine—while it had no effect on drugs not transported by P-gp such as SN-38, 5-FU, and methotrexate [64]. In renal carcinoma 786-O cells, elacridar used at higher concentrations (2.5 μM and 5 μM) in combination with sunitinib enhanced cytotoxicity by inhibiting P-gp activity [65]. Moreover, elacridar at 0.5 µM concentration improved sunitinib efficacy in P-gp and/or BCRP overexpressing colorectal cancer cell lines (HT29, COLO320, DLD1, and CACO2) as well as patient-derived organoids [66].

Thus, we and others observed a strong impact of elacridar at a concentration of 1 µM or lower (in most cases) on resistance to cytotoxic drugs that are P-gp substrates. In our study, we observed a stronger effect in the W1PR2 cell line with the highest *MDR1*/P-gp expression level, which suggests high effectiveness of elacridar in combating the resistance in cell lines with high P-gp expression level. A very similar effect was observed by our team previously [60]. This led us to investigate what is the mechanism of elacridar action in our model.

As a next step, we checked P-gp expression and activity in elacridar-treated cell lines. Although the protein expression following inhibitor treatment remained constant, there was a notable change in P-gp activity, which was confirmed both by flow cytometry and live-cell fluorescent microscopy, with Rho123 used as a fluorescent substrate for the protein. Similarly, we previously observed that elacridar inhibits the activity of another ABC protein—BCRP, without any changes in protein expression [35]. Thus, our results are consistent with the results of other investigators and suggest that the main mechanism of elacridar action is the inhibition of ABC transporters [27].

This indicates that elacridar successfully inhibits P-gp activity in our 2D cell culture model and is a good candidate for a drug that can increase the effectiveness of chemotherapy. However, a tumor is not composed of single cancer cells but is a complicated three-dimensional (3D) structure composed of cellular and non-cellular components that effectively limit drug diffusion, bind some anticancer drugs, and induce drug resistance in the CAM-DR mechanism [9,10,13,14].

Thus, we conducted similar experiments for the cells grown in the 3D model. First, we compared the expression of P-gp protein in the same cell lines growing as 2D monolayer and 3D spheroids, and we observed a very similar P-gp expression level in both cell culture conditions in W1PR1 and W1PR2 cell lines. In contrast, increased P-gp expression in 3D conditions was observed in W1PR1-C7 cell line. A similar level of *MDR1* transcript between cells growing in 2D and 3D cell culture conditions and expression of P-gp protein in 3D spheroids were observed by our team previously [54,67]. These results indicate that overexpression of P-gp protein is a universal feature of PAC-resistant cell lines, regardless of cell culture conditions.

Next, we performed a fluorescent microscopy analysis in 3D spheroids with Calcein-AM as a P-gp substrate. Calcein-AM is a non-fluorescent compound that undergoes hydrolysis in cells, turning into a fluorescent calcein [68]. Unlike Rho123, this compound becomes fluorescent once it is inside the cell, resulting in a significantly reduced background signal [69]. This is particularly important when working with spheroids, as they are fragile and often become damaged during washing off the excess substrate. The effect of elacridar on Calcein-AM accumulation in 3D spheres was similar to the effect on Rho123 accumulation in the 2D model. The W1 cell line with no P-gp expression retained the substrate. In contrast, the PAC-resistant cell lines did not retain the substrate, suggesting high P-gp activity in these spheroids. Very similar observations were made by us previously [60]. This experiment also indicates that Calcein-AM can easily diffuse into dense spheroids like spheroids formed by the W1 cell line, making it a good fluorochrome to study P-gp activity in a 3D cell culture model.

Next, we investigated the effect of elacridar on P-gp activity in 3D spheroids. No further increase in elacridar accumulation was observed in the drug-sensitive cell line. However, following elacridar treatment, every drug-resistant cell line showed increased substrate retention. The increase in Calcein-AM accumulation in 3D spheroids indicates that elacridar easily diffuses in the spheroid structure, and makes it a good candidate for increasing the effectiveness of chemotherapy based on P-gp substrates.

MTT tests carried out in the 3D model showed a few-fold increase in resistance to PAC in 3D cell culture conditions in comparison to 2D cell culture conditions in all three PAC-resistant cell lines. However, over a thousand-fold increase in PAC resistance was observed in the W1 cell line in 3D spheroids in comparison to a 2D monolayer. Elacridar treatment did not change the IC_50_ value in W1 3D spheroids. In contrast, elacridar was able to reverse PAC-resistance dramatically in PAC-resistant cell lines. Although the method chosen to determine the IC_50_ value is not perfect and allows for determining only approximate IC_50_ values due to the a very strong reduction in cell viability at a PAC concentration of 100 ng/mL in the presence of elacridar at a concentration of 1 µM, the obtained results indicate an effective decrease in the IC_50_ value in the presence of elacridar under 3D culture conditions in the resistant cell lines. However, the IC_50_ value in 3D spheroids was still over 20-fold higher than the IC_50_ value in the same cell line in a 2D monolayer following elacridar treatment. In summary, after elacridar treatment, the spheroids from drug-resistant cell lines were over 20-fold more resistant than the cells grown in a monolayer, and spheroids from W1 cell line were over a thousand-fold more resistant than the same cells grown in a monolayer. This indicates that other factors can be responsible for drug resistance in 3D spheroids. Looking at the structure of spheroids created from investigated cell lines, it is easy to distinguish two different kinds of spheroids. The regular, very dense spheroids that comprise the outer proliferation zone, inner quiescent zone and necrotic zone present in the biggest spheroids [70] were formed by W1 cells. In contrast, all PAC-resistant cell lines formed low-density spheroids. The very high increase in resistance of the W1 cell line between 2D and 3D cell culture conditions seems to result from the very high density of W1 spheroids, which effectively limits the diffusion of PAC, which is a big molecule with very limited diffusion properties [71,72,73,74]. This makes the high-density spheroids much more resistant to PAC action, as was observed by us previously [54,67] and is supported by studies of other investigators in breast cancer spheroids [75,76].

Another factor that can increase resistance to cytotoxic drugs is overexpression of extracellular matrix (ECM) molecules and expression changes in other drug-resistant genes, as it was observed by others [77] and by our team [74,78,79,80,81] in drug-resistant cell lines. It is widely recognized that the high expression of the ECM components in tumors restricts the penetration of therapeutic agents [82,83]. This effect is further intensified when certain cytotoxic drugs bind to cellular macromolecules, ultimately reducing the amount of drug available to target cancer cells [82]. Among the investigated drug-resistant cell lines, we previously observed a strong expression of *COL3A1* gene and its corresponding protein in the W1PR2 cell line, which seems to be related to PAC resistance [54,71].

Beyond acting as a physical barrier, the ECM also contributes to drug resistance through CAM-DR [82,83,84]. In this process, interactions between cancer cells and ECM proteins trigger intracellular signaling cascades and induce the expression of pro-survival genes, leading to increased resistance to drug-induced apoptosis [82,83]. This mechanism seems to be especially important in 3D spheroids, where cells are in close contact.

Increased intracellular signaling leading to increased expression of drug-resistant genes can also be related to decreased expression of some genes in drug-resistant cell lines. Previously, we observed decreased expression of some genes in PAC-resistant cell lines, among them: *SEMA3A* (Semaphorin 3 A) [79] and *PTPRK* (Receptor-type tyrosine-protein phosphatase kappa) [85]. *SEMA3A* is a tumor suppressor gene, and its decreased expression was observed in gastric and ovarian cancer and correlated with disease progression and poor prognosis [86,87]. In contrast, overexpression of SEMA3A correlated with inhibition of Src and FAK (focal adhesion kinase) kinases phosphorylation in tongue squamous cell carcinoma cell line SSC-9 [88]. PTPRK is a phosphatase, and its decreased expression was observed in breast cancer and correlated with the disease progression [89]. Mutation in the *PTPRK* gene correlated with chemoresistance in glioma [90]. Previously, we observed downregulation of PTPRK in fifteen ovarian cancer cell lines [85] and restoration of its expression by piperine treatment correlated with increased sensitivity to PAC [58]. Thus, the decreased expression of SEMA3A and PTPRK can be another factor that increases resistance in PAC-resistant spheroids.

Another aspect that should be discussed is the difference in the PAC response curves between spheroids formed from W1 and drug-resistant cell lines. In spheroids formed from PAC-resistant cell lines, we observed a two-step response curve in the absence of elacridar. These spheroids remained resistant even at very high PAC concentrations. Only when the PAC concentration exceeded the transport capacity of P-gp did cell death occur, which was reflected by a sudden collapse of the response curve. In contrast, after blocking of P-gp activity with elacridar, a sudden collapse of the response curve was observed at low PAC concentrations. However, some cells still survived, which was reflected by a flat response curve in the concentration range of 100 ng/mL to approximately 5000 ng/mL. Thus, about 50-fold increase in PAC concentration did not change spheroid viability. Furthermore, the level of viability seems to be related to spheroid density, being lowest in the W1PR1-C7 cell line (5–10% of control) and the highest in W1PR2 cell line (approximately 35% of control).

In contrast, in W1 spheroids, we observed a three-step response curve. At PAC concentration of 100 ng/mL, cell viability remained above 60% of the control, and did not change up to 2000 ng/mL (over a 20-fold increase). Only higher PAC concentrations were able to kill all cells within the spheroids.

These responsive curves appear to be associated with distinct cellular zones within the spheroids. In the outer zone proliferating cells are present, and this region is easily distinguishable in W1 spheroids. Since PAC is an antimitotic agent, it primarily kills proliferating cells. As most cells in drug-resistant spheroids are highly proliferative, blocking P-gp activity with elacridar results in PAC-induced cell death in these spheroids. In contrast, the inner zone contains quiescent cells that are much more resistant to PAC. This zone is considerably larger in W1 spheroids, which is reflected by their higher viability at increased PAC concentrations. However, very high PAC concentrations can also kill quiescent cells, likely through a different mechanism.

In conclusion, the conducted experiments suggest that elacridar, a dual P-gp and BCRP inhibitor, is a promising target for further research on combating MDR in ovarian cancer. The conducted research demonstrates its effectiveness in 2D and 3D in vitro conditions, by making the cells more susceptible to PAC and DOX. However, further research in more complicated models is necessary to further assess the effectiveness of elacridar as a drug in chemotherapy.

## 4. Materials and Methods

### 4.1. The Reagents and Antibodies

RPMI 1640 medium, fetal bovine serum (FBS), L-glutamine, amphotericin B (25 μg/mL), penicillin, streptomycin, trypsin EDTA solution, cell proliferation Kit I (MTT)—Thiazolyl Blue Tetrazolium Bromide, Calcein-AM (206700-1MG), and Rhodamine 123 (Rho 123) were obtained from Sigma (St. Louis, Missouri, USA). DPBS was bought from Corning (Corning, NY, USA). Cytotoxic drugs: PAC, CIS, DOX, and the inhibitor elacridar were sourced from Selleckchem (Houston, TX, USA). The anti-P-glycoprotein antibody (C219) (mouse monoclonal, Alx-801-002-c100) came from Enzo, (Farmingdale, NY, USA). The for GAPDH, β-actin, and goat anti-mouse horseradish peroxidase (HRP)-conjugated antibody were purchased from Proteintech, (Rosemont, IL, USA) (SA00001-1-A), and the fluorescent Alexa Fluor^®^488 AffiniPure™ Donkey anti-mouse IgG (H + L) (715-545-150, 1:200) was acquired from Jackson ImmunoResearch Laboratories (Cambridgeshire, UK). The RnaseOUT (10777019) and M-MLV reverse transcriptase kit (28025013) were obtained from Invitrogen by Thermo Fisher (Waltham, MA, USA). The materials used in the Western blot procedure (gels, protein marker, and membranes) were purchased from Bio-Rad (Bio-Rad Laboratories Ltd., Watford, Hertfordshire, UK).

### 4.2. Cell Culture

The W1 human primary ovarian cancer cell line was established in our laboratory using ovarian cancer tissue obtained from an untreated patient as described previously [91]. Paclitaxel-resistant sublines (W1PR1, W1PR1-C7, and W1PR2) were created by gradually exposing W1 cells to increasing concentrations of PAC, eventually reaching a final concentration of 1100 ng/mL. These cells were grown as a monolayer in RPMI 1640 medium supplemented with 10% (*v*/*v*) FBS, 2 mM L-glutamine, 0.1 mg/mL penicillin, 100 U/mL streptomycin, and 25 µg/mL amphotericin B, incubated at 37 °C in a 5% CO_2_ atmosphere.

### 4.3. RNA Isolation, cDNA Synthesis, and QPCR

RNA was extracted from all cell lines using the Gene Matrix Universal RNA Purification Kit (EURx, Ltd., Gdańsk, Poland) according to the manufacturer’s guidelines. The RNA concentration was determined by measuring absorbance at 260 and 280 nm. To synthesize cDNA, the reverse transcription was performed with 1.5 µg of RNA using the CFX Opus 96 Real-Time PCR System (Bio-Rad, Hercules, CA, USA). 1 μL oligodT18A (IBB PAN, Warsaw, Poland) and 1 μL dNTP Mix (ThermoFisher, Waltham, MA, USA, R0192) were added to each RNA sample, and denaturation followed at 65 °C for 5 min. Subsequently, 4 µL 5x First Strand Buffer, 2 µL DTT, 0.5 μL RnaseOUT, and 0.5 μL M-MLV RT were added. The reaction was carried out at 37 °C for 60 min, followed by 75 °C for 15 min.

Real-time PCR was conducted with the CFX Opus 96 Real-Time PCR System (Bio-Rad, USA). Each reaction sample contained: 1 μL cDNA solution, 12.5 μL Takyon™ ROX SYBR^®^ MasterMix blue dTTP (Eurogentec, Kaneka Eurogentec, Liège, Belgium), 1 μL of each sequence-specific primer (7.5 µM) from Oligo.pl (Warsaw, Poland) (Table 6) and 9.5 μL water free of nucleases. A negative control was also included, using water free of nucleases in place of cDNA.

The real-time PCR was carried out under the following conditions: an initial denaturation step at 95 °C for 15 min, followed by 45 cycles of denaturation at 95 °C for 15 s, primer annealing at 60 °C for 30 s, and extension at 72 °C for 30 s. A final extension step was performed at 72 °C for 30 s. Glyceraldehyde-3-phosphate dehydrogenase (GAPDH) served as the housekeeping gene (internal reference control).

The results were analyzed with the CFX Maestro software (Bio-Rad v2.3 (5.3.022.1030)). Gene expression was assessed through the relative quantification (RQ) method. Gene expression levels for each sample were assessed relative to the 2D culture condition, which served as the calibrator (RQ = 1). The relative quantification was determined using the formula: RQ = [expression in sample (3D condition)/expression in calibrator (2D condition)]. Data visualization was performed with SigmaPlot software, version 15.0 (Systat Software GmbH, Erkrath, Germany).

### 4.4. Two-Dimensional MTT Assay

All W1 cell lines at a concentration of 3000 cells per well were seeded into a 96-well plate in 200 µL of cell culture medium. After 48 h, the medium was replaced, and the cells were treated with elacridar (0 µM, 0.1 µM, or 1 µM) along with varying concentrations of PAC, DOX, or CIS drugs. Following a 72 h incubation period, an MTT assay was performed. The medium was replaced with 170 µL of fresh medium, and 100 µg of MTT reagent was added to each well, followed by a 1 h incubation. The resulting formazan crystals were dissolved in 200 µL of DMSO, and the absorbance was measured at wavelengths of 570 nm and 720 nm using the Synergy LX Multi-Mode Reader from BioTek Instruments, Inc. (Winooski, VT, USA) The IC_50_ values were then determined from the results.

### 4.5. Three-Dimensional MTT Assay

All W1 cell lines (15k cells per well) were seeded into non-adherent, 96-wells plates (BRAND plates inter Grade, F-bottom, 781902 (Merck, Darmstadt, Germany)) and maintained for 48 h. Following this initial incubation, 100 µL of the medium in each well was replaced with 100 µL of medium containing elacridar (final concentration: 0 µM, 0.1 µM, or 1 µM) together with the designated doses of PAC or DOX. Next, the spheroids were incubated for an additional 72 h. Cell viability was assessed using the MTT-based Cell Proliferation Kit I (Roche 11465007001). For the assay, 100 μL of medium was removed from each well, and replaced with 10 μL of the MTT labeling reagent. After a 4 h incubation, 100 µL of solubilization solution was added, and the plates were left overnight in the incubator to allow complete dissolution of the formazan crystals. Absorbance was measured (at 570 nm and 720 nm) using Synergy LX Multi-Mode Reader (BioTek Instruments, Inc.) IC_50_ values were calculated based on the absorbance data.

### 4.6. Immunofluorescence

Glass coverslips were placed into 24-well plates (one coverslip per well). Next, the cells were cultured in the wells at a density of 100,000 cells per well. Once cultures reached approximately 70% confluence, the cells were rinsed with phosphate-buffered saline (PBS), followed by fixation and permeabilization using a cold acetone/methanol mixture (1:1) for 15 min at room temperature (RT). After an additional PBS wash, nonspecific binding sites were blocked with 3% BSA for 30 min at RT. The samples were subsequently incubated with the primary antibody against P-gp (C219, 1:50) for 2 h at RT. After 3 PBS washes, the coverslips the Alexa Fluor^®^ 488-conjugated donkey anti-mouse secondary antibody for 1 h at RT. Following another series of PBS washes, the coverslips were mounted with a DAPI-containing medium (Sigma, F6057) for nuclei staining. P-gp protein expression was examined using a Zeiss Axio-Imager.Z1 fluorescence microscope (Oberkochen, Germany) and analyzed with Zen Blue v3.3 software.

### 4.7. Life-Cells Fluorescence (Rh123 Accumulation) (2D)

All cell lines were seeded into 24-well plates at a density of 1 × 10^4^ cells per well. After 72 h of growth, the cells were treated with the appropriate dosage of elacridar (final concentration 0.1 and 1 µM). After 1 h of incubation, the medium was changed to the medium with the addition of elacridar in proper concentration and Rh123 (1 µg/mL) and incubated 1 h. Next, the cells were washed three times with a cold solution of 50 µM Verapamil in PBS. Microscopic observation followed, and images were captured at 20× magnification for FITC and VIS channels (Leica DMi8 microscope, Leica, Germany).

### 4.8. Live-Cell Fluorescence (CA Accumulation) in 3D

The cells were seeded into non-adherent 96-well plates at a density of 5 × 10^4^ cells per well and cultured for 5 days. The medium was then replaced with medium containing the appropriate amount of elacridar, and the cells were incubated for 1 h. Next, the spheroids were treated again with the same concentration of elacridar, with 0.25 μM Calcein-AM added for 1 h. The spheroids were washed three times with a cold solution of 50 µM Verapamil in PBS and observed under a microscope (Leica DMi8 microscope, Leica, Germany) taken at 40× magnification for FITC and VIS channels.

### 4.9. Protein Isolation and Western Blot Analysis

Cells were washed three times with PBS containing Ca/Mg ions and lysed using RIPA buffer (300 µL for 1 × 10^6^ cells) with a protease inhibitor cocktail (Roche Diagnostics GmbH, Mannheim, Germany) for 80 min on ice at 4 °C. The lysates were then centrifuged at 13.4 × 10^3^ rpm for 30 min at 4 °C. Protein concentrations were measured using the Bradford protein assay system (Bio-Rad Laboratories, Hemel Hempstead, UK). Samples for electrophoresis were prepared from 15 μg of protein mixed with 4× loading buffer (Bio-Rad Laboratories, Hemel Hempstead, UK) containing 10% β-mercaptoethanol. Proteins were separated by SDS-PAGE on a 4–20% mini-PROTEAN^®^ TGX™ precast gel and transferred to a nitrocellulose membrane using the Trans-Blot^®^™ transfer system (Bio-Rad Laboratories, Hemel Hempstead, UK). The membrane was then blocked with 5% milk in TBS/Tween (0.1 M Tris-HCl, 0.15 M NaCl, 0.1% Tween 20) and incubated overnight with primary antibodies against P-gp (Enzo, Alx-801-002-c100, 1:400), followed by a 3 h incubation with HPR-labeled mouse secondary antibody (Proteintech, SA00001-1-A, 1:10,000). The same membrane was subsequently washed with TBS/Tween (0.1 M Tris-HCl, 0.15 M NaCl, 0.1% Tween 20) for 1 h and incubated with the primary antibody against the reference protein, either β-actin (1:7500) or GADPH (1:40,000), followed by a 3 h incubation with HRP-conjugated secondary antibody. Signals were visualized using a chemiluminescence detection system (ECL, Femto Super Signal Reagent) and ChemiDoc™ (Bio-Rad Laboratories, Hemel Hempstead, UK).

### 4.10. Flow Cytometry Analysis

P-gp efflux function after elacridar exposure was evaluated using Rhodamine 123 (Rho123) fluorescence. Cells were suspended at 1 × 106 cells/mL in culture medium and incubated with 0.1 or 1 µM elacridar for 1 h at 37 °C and mixed at 800 rpm. Rho123 was then added to a concentration of 1 µg/mL, and the samples were incubated for an additional hour under the same conditions. After incubation, the cells were placed on ice, centrifuged at 200× *g* for 5 min at 4 °C, and washed twice with ice-cold PBS supplemented with 50 μM verapamil, and immediately analyzed on a MACSQuant X flow cytometer (Miltenyi Biotec GmbH, Cologne, Germany). A total of 10,000 events were collected per sample, and fluorescence was detected at 488 nm. Data analysis was performed with FlowJo software (v10.9.0).

## Figures and Tables

**Table 1:** Summary of the sensitivity of each cell line to PAC, DOX, and CIS under 2D cell culture conditions. The IC_50_ values for PAC, DOX, and CIS are shown for all tested lines, with the paternal W1 cell line normalized to a reference value of 1. Fold changes in resistance relative to W1 are underlined. Upward and downward arrows indicate increases or decreases in IC_50_ values compared to W1. Statistical significance is marked as ** *p* < 0.01 and *** *p* < 0.001.

Cell Line	PAC IC_50_ (ng/mL)	DOX IC_50_ (ng/mL)	CIS IC_50_ (ng/mL)
W1	3.18 (2.77–3.98) 1	32.07 (18.3–69.0) 1	19,741 (9323–26,667) 1
W1PR1	1960 (1871–2062) 616 ↑ ***	6904 (3303–13,720) 215 ↑ ***	21,566 (18,033–28,537) 1.09 ↑
W1PR1-C7	1199 (934–1433) 377 ↑ ***	2769 (1139–4446) 86 ↑ **	14,118 (9751–16,974) 1.40 ↓
W1PR2	3384 (1995–4340) 1064 ↑ ***	7836 (5674–9938) 244 ↑ **	16,779 (13,826–19,986) 1.17 ↓

**Table 2:** Summary of PAC resistance levels across the tested cell lines under 2D culture conditions, comparing PAC treatment alone with PAC administered alongside elacridar at 0.1 and 1 µM. The IC_50_ values for PAC are shown for each line, with resistance under PAC-only conditions normalized to a value of 1 for each cell line. Underlined values represent the fold increase in sensitivity observed upon elacridar co-treatment relative to PAC alone. Downward arrows indicate decreases in IC_50_ values compared with the control condition. Statistical significance *** *p* < 0.001.

Cell Line	Control PAC IC_50_ (ng/mL)	Elacridar 0.1 µM PAC IC_50_ (ng/mL)	Elacridar 1 µM PAC IC_50_ (ng/mL)
W1	3.18 (2.77–3.98) 1	2.70 (1.99–3.76) 1.18 ↓	2.65 (1.92–3.69) 1.20 ↓
W1PR1	1960 (1871–2062) 1	4.86 (3.34–6.34) 404 ↓ ***	3.23 (1.82–5.02) 607 ↓ ***
W1PR1-C7	1199 (934–1433) 1	2.29 (1.08–3.48) 538 ↓ ***	1.93 (0.98–3.17) 620 ↓ ***
W1PR2	3384 (1995–4340) 1	5.20 (3.34–7.48) 650 ↓ ***	3.58 (3.02–5.02) 945 ↓ ***

**Table 3:** Summary of DOX resistance levels in 2D-cultured cell lines treated with DOX alone or in combination with elacridar at 0.1 and 1 µM. IC_50_ values for DOX are listed for each cell line. For every line, DOX resistance under DOX-only condition was normalized to a value of 1. Underlined values indicate the fold increases in sensitivity observed when elacridar was added compared with DOX alone. Downward arrows indicate reduced IC_50_ values relative to the control. Statistical significance: ** *p* < 0.01, *** *p* < 0.001.

Cell Line	Control DOX IC_50_ (ng/mL)	Elacridar 0.1 µM DOX IC_50_ (ng/mL)	Elacridar 1 µM DOX IC_50_ (ng/mL)
W1	32.07 (18.3–69.0) 1	25.0 (16.5–48.7) 1.28 ↓	21.6 (15.6–32.6) 1.48 ↓
W1PR1	6904 (3303–13,720) 1	84.6 (36.6–210) 82 ↓ ***	45.5 (16.2–84.3) 152 ↓ ***
W1PR1-C7	2769 (1139–4446) 1	36.1 (17,6–49.8) 77 ↓ **	28.4 (21.8–34.1) 98 ↓ **
W1PR2	7836 (5674–9938) 1	63.8 (42.5–81.4) 123 ↓ **	18.0 (5.67–32.0) 436 ↓ **

**Table 4:** Summary of CIS-resistance in 2D-cultured cell lines treated with CIS alone or in combination with elacridar at 0.1 and 1 µM. IC_50_ values for CIS are indicated for each cell line, with CIS resistance under CIS-only conditions normalized to a value of 1. Underlined values indicate the fold increases in sensitivity observed with elacridar treatment compared to the CIS alone. Upward and downward arrows indicate increases or decreases, respectively, in IC_50_ relative to the control condition.

Cell Line	Control CIS IC_50_ (ng/mL)	Elacridar 0.1 µM CIS IC_50_ (ng/mL)	Elacridar 1 µM CIS IC_50_ (ng/mL)
W1	19,741 (9323–26,667) 1	16,355 (13,462–17,921) 1.21 ↓	12,752 (8999–16,126) 1.55 ↓
W1PR1	21,566 (18,033–28,537) 1	20,099 (15,600–25,505) 1.07 ↓	22,470 (17,658–28,079) 1.04 ↑
W1PR1-C7	14,118 (9751–16,974) 1	14,906 (11,404–18,409) 1.06 ↑	14,998 (12,429–17,808) 1.06 ↑
W1PR2	16,779 (13,826–19,986) 1	20541 (14,303–24,763) 1.22 ↑	21,291 (16,666–28,983) 1.26 ↑

**Table 5:** Summary PAC resistance in 3D-grown cell lines treated with PAC alone or PAC combined with elacridar at a concentration of 1 µM. PAC IC_50_ values are presented for each cell line, with resistance under PAC-only treatment normalized to a value of 1. Underlined values indicate the fold increase in sensitivity observed with elacridar co-treatment. Upward and downward arrows indicate increases or decreases in IC_50_ relative to PAC alone. Statistical significance ** *p* < 0.01.

Cell Line	Control PAC IC_50_ (ng/mL)	Elacridar 1 µM PAC IC_50_ (ng/mL)
W1	4539 (3486–6211) 1	4597 (3690–6220) 1.01 ↑
W1PR1	8405 (4788–13,305) 1	65.3 (56.4–74.3) 129 ↓ **
W1PR1-C7	4187 (3797–4540) 1	53.0 (52.0–54.1) 79.0 ↓ **
W1PR2	5894 (4875–6337) 1	78.3 (75.1–84.2) 75.3 ↓ **

**Table 6:** Primers used in the Q-PCR reaction.

Transcript	Sequence (5′−3′ Direction) Forward	Sequence (5′−3′ Direction) Reverse	ENST Number http://www.ensembl.org, Accessed on 11 December 2025	Product Size (bp)
*MDR1*	TGACAGCTAC- AGCACGGAAG	TCTTCACCTC- CAGGCTCAGT	00000265724	131
*GAPDH*	GAAGGTGAAG- GTCGGAGTCA	GACAAGCTTC- CCGTTCTCAG	00000229239	199

## Data Availability

The data presented in this study are openly available in [https://repod.icm.edu.pl/dataset.xhtml?persistentId=doi:10.18150/LLAXWZ], accessed on 11 December 2025 at [https://doi.org/10.18150/LLAXWZ], accessed on 11 December 2025.
